# Vein of Galen Aneurysmal Malformation: A Case Report and Literature Review

**DOI:** 10.7759/cureus.51305

**Published:** 2023-12-29

**Authors:** Khushi Bhattarai, Marmik Patel, Monica Garcia, Florentina Litra

**Affiliations:** 1 Pediatrics, University of Florida, Pensacola, USA; 2 Pediatrics, Ascension Sacred Heart, Pensacola, USA

**Keywords:** endovascular embolisation, transvenous embolization, congenital vascular malformation, extra cardiac shunting, vein of galen malformation

## Abstract

The vein of Galen aneurysmal malformation (VGAM) is a rare congenital vascular malformation caused by the maldevelopment of its embryonic precursor, the median pros encephalic vein of Markowski. Although most of the VGAM cases are diagnosed in the neonatal period, sometimes it can also present during early childhood. It is very crucial to intervene immediately following the diagnosis because if left untreated, morbidity and mortality are imminent. The most common causes of morbidity and mortality are high-output congestive heart failure (most common neonatal presentation), hydrocephalus (most common presentation in infants), headache, and seizures.

We are presenting the case of a two-year-old male with global developmental delay, failure to thrive, and macrocephaly who presented with recurrent generalized tonic-clonic seizures. MRI/magnetic resonance venography (MRV)/magnetic resonance angiography (MRA) brain showed an enlarged vein of Galen with venous hypertension and aqueduct stenosis.

Treatment intervention included trans-arterial embolization of the right pericallosal, right/left lateral posterior, and medial posterior choroidal feeders with coils. The patient has had significant improvement in his neurocognitive functions including significant improvement in his speech/language development with outpatient therapies in between embolization.

## Introduction

Vein of Galen aneurysmal malformation (VGAM) is a congenital vascular malformation consisting of multiple arteriovenous shunts draining into the median pros-encephalic vein of Markowski, which is not actually the vein of Galen itself, but rather a persistent embryonic structure that precedes the vein of Galen [1].^ ^The arterial supply can arise from anterior and posterior circulation. It was first considered an aneurysm due to observed dilation in the vein of Galen but now it is preferred to call it VGAM since it is not a true aneurysm and malformation involving the precursor of vein of Galen, i.e., fetal median prosencephalic vein [2]. Their incidence is about one in three million population [3-5], and they represent less than 1% of cerebral AV malformation. The majority of VGAM become symptomatic in the neonatal period and if left untreated have an almost 100% morbidity and mortality [6]. The optimal management of VGAM hinges on a correct understanding of the anatomy and pathophysiology of these entities and the treatment of choice is transarterial embolization [2], although each case is unique.

Here we present a case of a three-year-old male child with developmental and cognitive delay, who presented with seizure-like activity, macro crania, hyperdynamic circulation with left ventricular hypertrophy (LVH), and left ventricular enlargement (LVE), and was found to have VGAM and the challenges faced during multi-staged transarterial embolization.

## Case presentation

A two-year-old male was referred from an outside hospital for altered sensorium and concern about the seizure activity for 45 minutes. He had a similar episode in the Emergency Room (ER) lasting two minutes for which he received a loading dose of levetiracetam, after which the seizure was aborted, but he remained altered. He was admitted to the medical unit for further management. Basic labs were normal. 

Physical examination revealed an asthenic body habitus with enlarged head of about 51 cm (>95% for age). He had a systolic murmur all over the precordium without thrill and heave, radiating to the carotid artery bilaterally. Cardiac examination was suggestive of hyperdynamic circulation with wide pulse pressure. He was altered in the ER but was back to his baseline after transfer to the medical unit with a reassuring neurological examination. During history taking, his parents mentioned that he had mild global developmental delay including speech delay. On further investigation, it was found that he had a similar episode of unresponsiveness lasting about one hour, six months back. At the time, he was evaluated in an outside ER with EEG which was normal, and echocardiography which showed LVE and LVH with normal ejection fraction. During that time, further workup including genetic testing was done for cardiomyopathy but later was lost to follow-up. 

**Figure 1:**
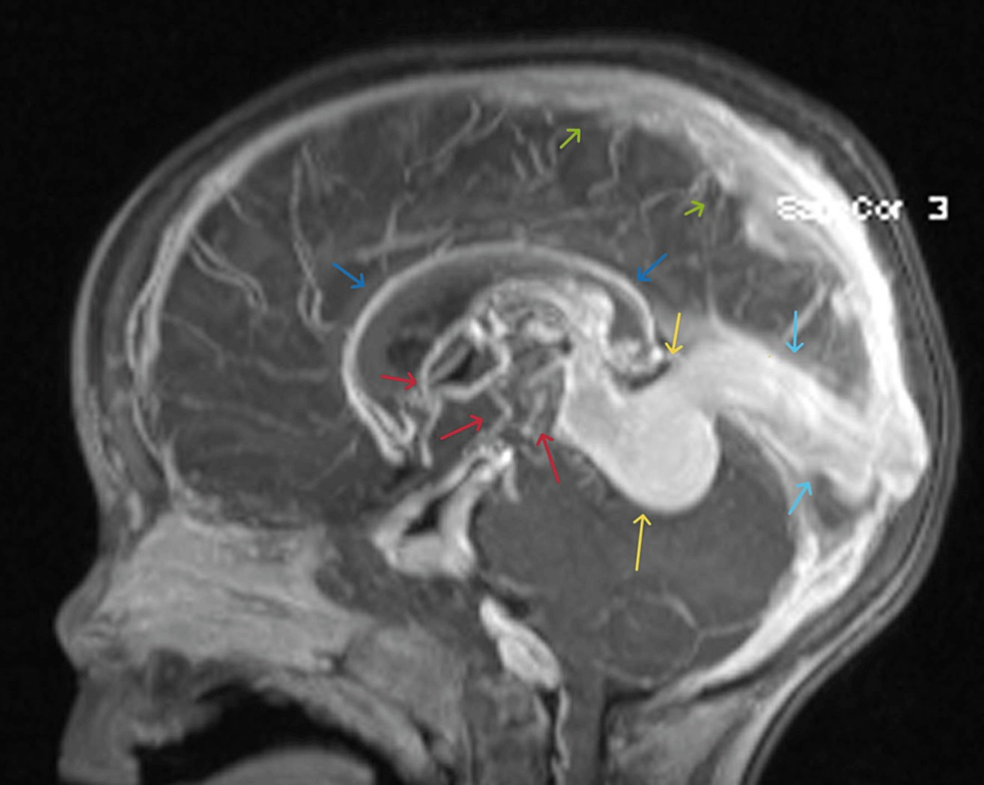
Arterial feeders (red arrows) arising from branches of the PCAs, thalamoperforating, choroidal, and pericallosal arteries. Venous drainage through the internal cerebral veins, enlarged vein of Galen (yellow arrows), enlarged straight (dark blue arrows) and superior sagittal sinuses (light blue arrow), and bilateral transverse sinuses (green arrows). Enlarged vein of Galen, measuring 1.9 cm in TR dimension and 3.7 cm in CC dimension. PCA: posterior cerebral artery; CC: craniocaudal

Due to these findings on history and physical exam, a CT brain was ordered to rule out any emergent condition. He was found to have an amorphous mass in the pineal area suggestive of a pineal tumor. EEG was found to be normal. Echocardiography showed mild LVH with moderate LVE with normal left ventricular ejection fraction (LVEF) and concern for extracardiac shunting. MRI/magnetic resonance venography (MRV)/magnetic resonance angiography (MRA) Brain/Spine/Neck were ordered at the earliest and was found to have a cluster of abnormal flow voids in the region of pineal/superior cerebellar region, an enlarged vein of Galen, consistent with VGAM (median prosencephalic arteriovenous fistula), arterial feeders arising from the branches of posterior cerebral arteries, thalamic-perforating, choroidal and pericallosal arteries, venous drainage through the internal cerebral veins, enlarged and patent vein of Galen and enlarged straight and superior sagittal sinuses and bilateral transverse sinuses. The nidus measured approximately 3.3 x 3.5 x 2.5 cm. Cerebral volume loss with mild to moderate hydrocephalus with no encephalomalacia or calcification was noted. Superior sagittal sinus, torcula, bilateral transverse and sigmoid sinuses, bilateral jugular bulbs, internal cerebral veins, the vein of Galen, and straight sinus were patent. The enlarged vein of Galen measured 1.9 cm in transverse dimension and 3.7 cm in craniocaudal dimension(Figure 1). Neurosurgery was consulted immediately and planned for the multi-stage transarterial embolization after the angiography to underline the angioarchitecture. 

**Figure 2:**
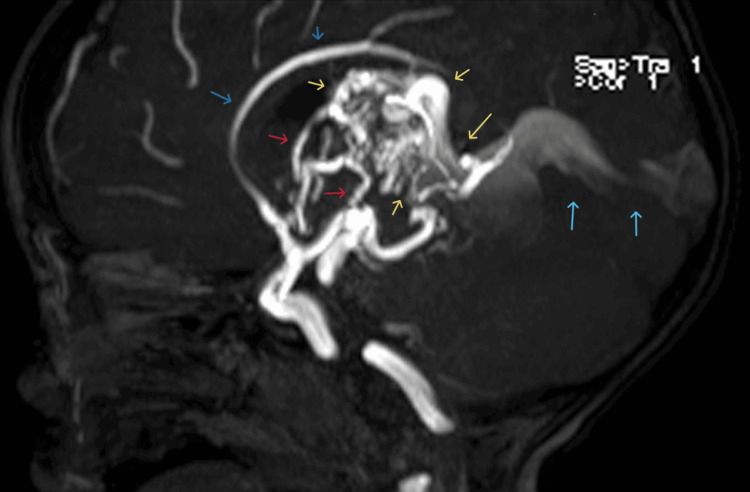
Vein of Galen aneurysmal malformation (yellow arrow). Status post transarterial embolization of the left lateral posterior choroidal artery feeders on April 5, 2022. The nidus has remained similar in size, since December 27, 2021. Arterial feeders (red arrows) as before, arising from branches of the PCAs, thalamoperforating, choroidal, and pericallosal arteries. Venous drainage as before, through the internal cerebral veins, enlarged vein of Galen (yellow arrow) and enlarged straight (blue arrow) and superior sagittal sinuses, and bilateral transverse sinuses. PCA: posterior cranial artery

The staging of transarterial embolization was dependent on the angioarchitecture found in the angiography. The patient underwent angiography and first endovascular transarterial embolization of the right pericallosal artery feeder with n-butyl-cyanoacrylate (n-BCA), with/without Guglielmi detachable coil (GDC). The second stage was planned six months later for embolization of the right lateral posterior choroidal and medial posterior choroidal feeders with GDC coils and n-BCA. Later, the third stage embolization of a fetal left posterior cerebral artery feeder with n-BCA was done three months later (Figure 2). During the fourth stage of embolization, neurosurgeons were unable to occlude the feeder with coils of multiple sizes due to the high flow of the targeted fistula precluding a safe embolization attempt of the right posterior cerebral artery feeder. Due to the need for an alternative plan, a combined transarterial and transvenous approach was used, in which the vein of Galen was temporarily occluded by the transvenous approach to decrease the flow. With this approach, the patient successfully underwent the fifth stage embolization of the right posterior cerebral artery choroidal feeder with GDC coils and n-BCA with the help of temporary occlusion of the vein of Galen by trans-venous catheterization through the femoral vein. The patient tolerated the procedure very well. Currently, the patient is showing significant clinical improvement in his appetite along with speech and cognitive functions. He did not experience further seizure activity, and his follow-up echocardiology showed improvement in LV dilatation. 

## Discussion

VGAM is a congenital vascular malformation consisting of multiple arteriovenous shunts draining into the median prosencephalic vein of Markowski, which is not the vein of Galen itself, but rather a persistent embryonic structure that precedes the vein of Galen [1]. During weeks 6-11 in utero, the median prosencephalic vein is responsible for venous drainage from the developing fetal choroidal plexus. As the typical embryological course continues, the vein of Markowski would eventually regress and the internal cerebral vein would take shape and continue drainage of the growing choroid plexus [7]. However, in the case of VGAM, arteriovenous shunt develops within the median prosencephalic vein instead. The shunt leads to an increase in blood flow, making the median proencephalic vein continuously dilated and, thus, it persists and the regression that would have taken place is effectively prevented. The sequel is an abnormal anatomy of the dural venous sinus due to increased vasculature leading to the post-natal VGAM [3]. 

Although VGAM develops in utero, signs and symptoms occur after birth when the low-resistance placental circulation is removed. VGAM most often manifests as high-output cardiac failure due to an increase in venous return to the right side of the heart [8]. The symptom severity depends on the underlying angioarchitecture of the VGAM as well as the age of the child [1]. The larger the shunt, the more the preload to the heart, and the earlier the child presents with the symptoms of heart failure. Patients with delayed presentation have smaller shunts; thus, it can present as mild cardiac symptoms like volume overload, tachycardia, and signs of cardiomegaly along with feeding difficulties and failure to thrive due to cardiac decompensation [1]. 

VGAM also causes hydrocephalus due to aqueduct compression from dilated VGAM, seizure, and developmental delay secondary to long-standing cerebral venous congestion and abnormal CSF flow. Sometimes in the neonatal period, the tremendous flow of blood to the fistula along with high cerebral venous pressure can lead to ischemic damage and cerebral edema. This causes rapid loss of brain tissue, which is referred to as melting brain, the most dreaded complication of VGAM [1]. 

Ultrasound of the head is the first line of investigation for suspected VGAM in neonates, but it's less sensitive in children where fontanel is closed. CT scan and MRI brain helps delineate the anatomy before the treatment, but it doesn’t give full angioarchitecture details of the pathology. MRA/MRV helps plan the steps before the endovascular procedure for the exact arterial and venous structure, but angiography is both the therapeutic and diagnostic gold standard.

Currently, endovascular embolization is the treatment of choice in patients with VGAM. Before the development of endovascular embolization, a newborn with VGAM had a nearly 100% mortality [1]. Recently Lajuana et al. reported 10.6% general mortality in patients undergoing endovascular embolization in a series of 233 patients, and only three deaths were due to the embolization procedure and the rest were due to the poor prognosis before the procedure [9]. Also, 74% of survivors were neurologically normal on follow-up. The patient’s clinical presentation dictates the timing of the procedure. Routinely, the procedure is scheduled around five to six months of age in case of non-emergency. The goal is to prevent the development of cerebral venous hypertension and heart failure. It involves multiple stages that target the different pedicles to avoid parenchymal bleeding and venous thrombosis. Congestive heart failure refractory to medical management necessitates emergency embolization to relieve the hemodynamic load on the heart [1]. 

Endovascular access can be gained via a transarterial or transvenous route. The embolic glue of choice to occlude the arteriovenous fistula on the arterial side is N-butyl-cyanoacrylate, and more recently onyx has been used [1,10]. Detachable micro-coils can also be used but their application may take longer time and have a higher risk of rupture [1,10]. Some surgeon prefers the venous route but others reserve it for patients in whom the arterial route is difficult or after the failure of the transarterial procedure, as occlusion of venous aneurysm may hinder deep venous drainage or result in perforation of the venous aneurysm [1]. 

Our patient needed multiple stages for decreasing the flow through the VGAM by occluding the feeding artery. The patient tolerated the procedure well and after the second stage, improvement in the neurodevelopmental outcome started to be noticed. After the final stage, there was significant neurodevelopmental improvement along with improvement in the weight gain and stabilization of the head circumference, which was also appreciated by the parents. 

## Conclusions

VGAM, although rare, is the most common intracranial vascular malformation in children. It is usually diagnosed in the prenatal period, but some cases are diagnosed late in infancy and childhood. In children, it can manifest with seizures, developmental delay, hydrocephalus, and failure to thrive. When a child presents with the above features, along with LVH and LVE with preserved ejection fraction on echocardiology, extracardiac shunting like AV malformation should be considered. Echocardiology findings can be confused with dilated cardiomyopathy and can delay the diagnosis, as in our case. MRI is more sensitive than a CT scan for the diagnosis of VGAM, and MRA/MRV should be done along with MRI when VGAM is suspected, to identify the type and number of feeders and the patency of the preceding venous drainage, along with the hydrocephalus. 

Catheterization has diagnostic and therapeutic benefits, as it is important to know the angioarchitecture before the embolization procedure. The mainstay treatment is transarterial embolization with glue and/or coils. It can require multiple staged procedures depending on the number of feeder arteries supplying the pros encephalic vein. With age, the velocity of blood flow into feeders increases and it makes the procedure difficult and might need alternative strategies like combined arterial and venous approach. The final goal is the complete obliteration of the malformation with normal development, without focal neurological deficits. Traditionally, VGAM had a poor prognosis, but endovascular embolization has done well in improving the neurological outcome. 
